# Hepatic sonic hedgehog protein expression measured by computer assisted morphometry significantly correlates with features of non-alcoholic steatohepatitis

**DOI:** 10.1186/s12876-019-0951-y

**Published:** 2019-02-11

**Authors:** Michael Estep, Rohini Mehta, Gary Bratthauer, Lakshmi Alaparthi, Fanny Monge, Simon Ali, Dinan Abdelatif, Zahra Younoszai, Maria Stepanova, Zachary D. Goodman, Zobair M. Younossi

**Affiliations:** 1 Center for Liver Diseases, Department of Medicine, Inova Fairfax Hospital, Virginia, USA; 2 Betty and Guy Beatty Center for Integrated Research, Claude Moore Health Education and Research Building, Inova Health System, 3300 Gallows Road, Falls Church, VA 22042 USA; 3 Center for Outcomes Research Liver Diseases, Washington, DC USA

**Keywords:** NAFLD, Ballooning degeneration, NASH, Hedgehog

## Abstract

**Background:**

Hepatic expression of Sonic Hedgehog (SHH) is associated with Non-alcoholic fatty liver disease (NAFLD) and development of Non-alcoholic steatohepatitis (NASH). Hepatic SHH detection increases with the diagnosis of NASH. This pilot study was designed to confirm that staining for SHH is useful in NASH diagnosis and determine whether quantification of staining by computer assisted morphometry (CAM) can be used to assess severity of ballooning degeneration.

**Methods:**

SHH was detected by immunohistochemistry (IHC) on paraffin-embedded liver sections in subjects (*N* = 69) with biopsy proven NAFLD and no liver disease (control). Serum samples were also available for these subjects. Post-staining, a digitized image of the section was acquired and an area quantification algorithm was used to quantify the degree of SHH expression. Additionally, circulating M30, M65, and SHH were measured by ELISA.

**Results:**

Notably, hepatic SHH expression correlated with histologic ballooning degeneration (rho = 0.62, *p* < 0.0001), steatosis grade (rho = 0.554, *P* < 0.001), Mallory-Denk bodies (rho = 0.54, P < 0.001), pericellular fibrosis (rho = 0.527, P < 0.001), and lymphocytic infiltration (rho = 0.435, *P* < 0.0002). Additionally, hepatic SHH expression correlated with circulating M65 (rho = 0.588, p < 0.0001), and circulating M30 (rho = 0.375, *p* = 0.001), as well as AST and ALT (rho = 0.43, *p* = 0.0004, and rho = 0.27, *p* = 0.03, respectively). Further, serum M30 was almost twice as high in NASH patients compared to non-NASH (539.1 ± 290.8 U/L vs. 287.6 ± 190.5 U/L; *p* = 0.0002), while M65 was almost three times higher in NASH patients compared to non-NASH (441.2 ± 464.2 U/L vs. 162.8 ± 353.1 U/L, *P* = 0.0006). Logistic modeling indicates hepatic SHH expression and presence of type 2 diabetes as independent predictors of advanced fibrosis (defined as portal and pericellular fibrosis > 2: OR = 1.986, *p* = 0.01, and OR = 3.280, *p* = 0.03, respectively).

**Conclusion:**

Thus, our findings show quantitation of SHH expression by CAM can provide a tool for quantifying changes in hepatocyte injury and assist in unambiguous staging/grading of NASH. Our study showed minimal interobserver variability using CAM based quantification. Once validated, CAM assessment of hepatic SHH could benefit clinical trials or long term outcomes studies of NASH subjects.

## Background

Non-alcoholic steatohepatitis (NASH) is part of the spectrum of non-alcoholic fatty liver disease (NAFLD) [1]. Definitive diagnosis of NASH requires a liver biopsy and is established based the minimum criteria of 5% of tissue with fat (steatosis); presence of lobular inflammation; and hepatocellular injury termed “ballooning degeneration” [2]. When evaluating for this diagnosis, histologic assessment of steatosis and inflammatory cell quantification is relatively straight forward [3], leading to minimum of variation in scoring [4]. Ballooning degeneration, on the other hand can be subtle and difficult to detect and quantify leading to significant inter-observer inconsistency [5–7].

The difficulty in assessing ballooning degeneration arises from its variable presentation, combined with a largely descriptive definition that lack consensus regarding underlying pathogenesis [8]. Ballooned hepatocytes are typically large round cells with a reticulated cytoplasm on hematoxylin and eosin (HE)-stained sections [9]. Specifically, hepatocyte ballooning degeneration is characterized by visible swelling of the hepatocyte and vacuolization with clear cytoplasm. In some cells, CK18 intermediate filament loss accompanies ballooning [9]. However, the morphological features of ballooning degeneration can be mimicked by glycogenated hepatocytes or microvesicular fatty changes in hepatocytes [10].

NASH has been considered the progressive form of NAFLD [1, 2]. In this context, most of the therapeutic clinical trials have focused on identifying patients with NASH [11]. On the other hand, severity of hepatic fibrosis has been shown to determine the long-term outcome of NAFLD [4, 12–14]. Nevertheless, because ballooning degeneration is independently associated with hepatic fibrosis [15], it seems likely that the type of hepatocellular injury that results in ballooning may simultaneously stimulate fibrogenesis. Consequently, a histologic stain that reliably identifies mild forms of ballooning that is typically difficult to identify with routine staining would be of great help to establish the diagnosis of NASH. Furthermore, quantification of the degree of staining for ballooning degeneration may be helpful for assessing the severity of NASH.

A possible candidate for identification and quantification of hepatocyte ballooning is detection of hepatocyte Sonic Hedgehog signaling protein (SHH). In a study by Guy et al. [16], qualitative assessment of SHH signal by IHC in liver biopsies correlated with the diagnosis of NASH as well as response to therapy. In the current investigation immunostained hepatic SHH was quantified using computer assisted morphometry (CAM). The pilot study assessed whether SHH quantity indicates severity of the disease as determined by circulating cytokeratin 18 (M65), its caspase degradation product (M30) which have been shown to correlate with histologic severity in NAFLD [17, 18] as well as pathologic evaluations.

## Methods

### Subjects

Patients with biopsy proven NAFLD and control subjects with no or minimal histologic abnormalities were included in the study (*N* = 69). The biopsies were evaluated by single hepatopathologist. Fasting serum samples that had been collected within 3 months of the biopsy were available along with clinical and laboratory data. Biopsies were a mixture of needle core and wedge, with the needle core having an average length of 18.8 mm. Exclusion criteria included inadequate biopsy, excessive use of alcohol, history of viral hepatitis or other liver diseases. All patients participating in this study were pre-consented in accordance with our institutional review board.

### Measurement of circulating Analytes

Using fasting serum, circulating analytes [M30 Apoptosense® CK18 Kit (DiaPharma, OH) and M65 EpiDeath® ELISA (DiaPharma, OH)] were measured by ELISA. Circulating SHH concentrations were measured by ab100639 – Sonic Hedgehog Human ELISA Kit (Abcam, MA). All protocols were performed as per manufacturers’ instructions.

### Immunohistochemistry and histologic assessment

Histologic assessment included semiquantitative scoring of the following parameters: steatosis (estimated proportion of parenchyma occupied by fat) on a scale of 0–4 with 0 = none, 1 = > 0 but < 5%, 2 = 5–33%, 3 = 34–66%, 4 = > 66%; lobular and portal inflammation, and features of hepatocellular injury (apoptosis, ballooning, Mallory-Denk bodies) on a scale of 0–3 with 0 = none, 1 = mild or few, 2 = moderate, 3 = marked or many. Components of fibrosis were graded separately with centrilobular pericellular/perisinusoidal fibrosis typical of early stage NAFLD and portal fibrosis scored from 0 to 3 for none, mild, moderate and marked. Pre-cirrhotic bridging fibrosis was scored as 0 (absent), 1 (few = bridges linking fewer than 50% of central veins and portal tracts), or 2 (many = linkage of greater than 50% of central veins and portal tracts. Cirrhosis was scored as 0 (absent), 1 (early or incomplete) or 2 (established or advanced). Most cirrhotic liver biopsies also had portal fibrosis scores of 3, since the portal tracts were typically incorporated into the cirrhotic septa.

Immunohistochemical staining for SHH was done by standard procedure using anti-SHH antibody (Abcam, MA; ab53281) at a dilution of 1:4000. Briefly, following deparaffinization and rehydration, epitope retrieval was performed in a steamer by incubating slides in IHC-TEK Epitope retrieval solution (IHCWorld, MD) for 40 min. After rinsing, slides were blocked using 3% H_2_O_2_ for 30 min, then rinsed again. Primary antibody was applied and slides were allowed to incubate for 60 min in a humidified chamber. Following successive wash steps, secondary antibody was applied and allowed to incubate for 30 min (EnVision™ + Dual Link System-HRP, Dako, CA). ImmPACT DAB (Vector Laboratories, CA) was used for detection, and slides were counterstained with hematoxylin before dehydration and mounting.

Post-staining, a digitized image of the entire section was acquired using an Aperio/Leica Scanscope XT scanner at 40X magnification. Twelve annotations of equal area consisting of typical hepatic parenchyma were chosen at random and distributed throughout the biopsy; placement of these areas of quantification was done by two separate researchers working independently to assess methodological robustness. An area quantification algorithm was then used to quantify the degree of SHH expression in these annotated areas.

### Statistical analysis

Clinico-demographic and laboratory parameters were compared between subjects with and without NASH using chi-square test (for categorical parameters) or Mann-Whitney test (for continuous parameters). Correlations between continuous parameters were calculated using Spearman’s non-parametric approach. Independent predictors of NASH and advanced fibrosis in patients with NAFLD were assessed using multiple logistic regression models with bidirectional stepwise selection of parameters; only those with *p* < 0.05 were left in the models. All analyses were run in SAS 9.3 (SAS Institute, Cary, NC).

## Results

### Cohort characteristics

**Fig. 1:**
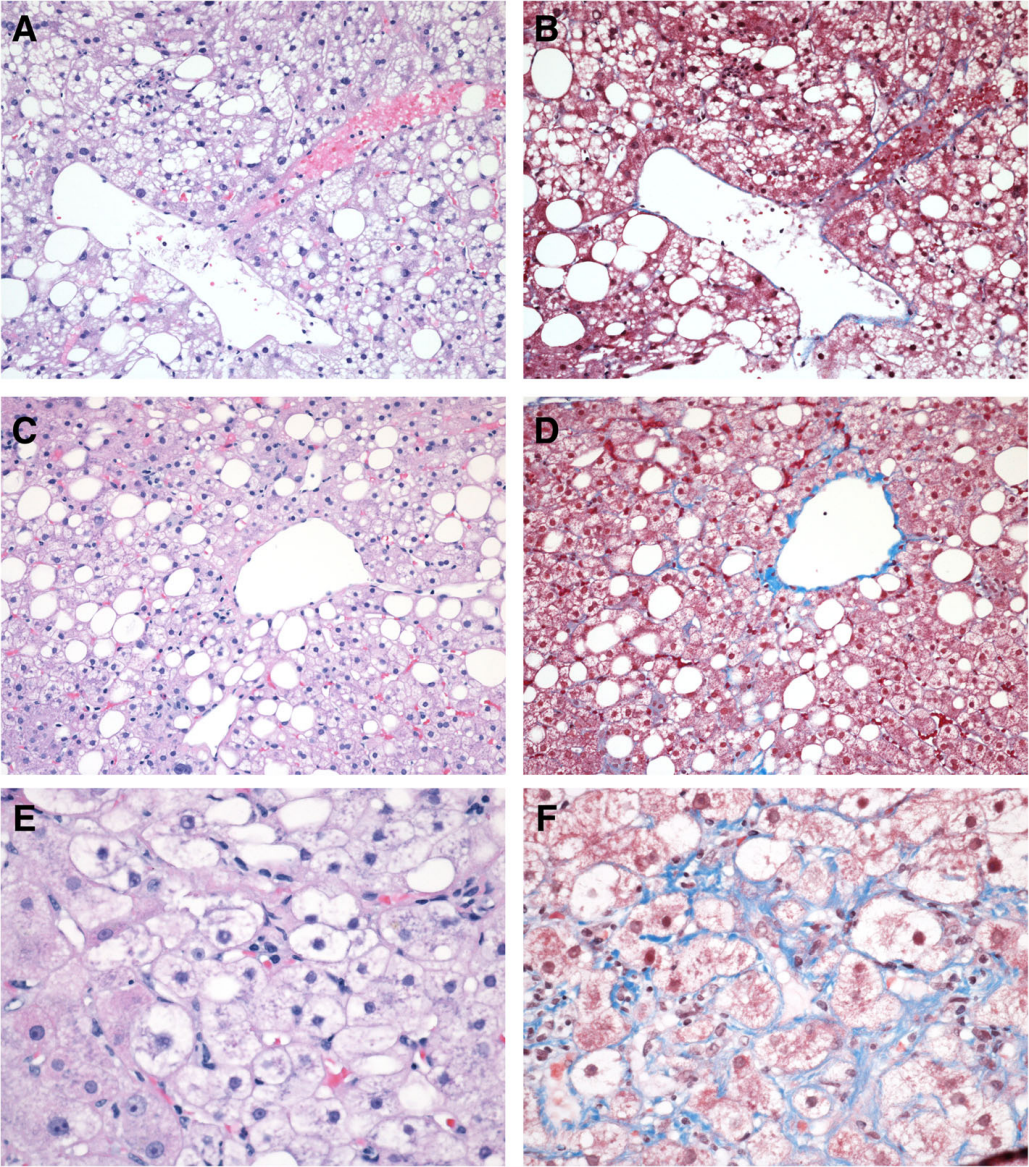
Representative Masson trichrome and H&E stained liver biopsies of NALFD, “borderline” NASH, and NASH: **a**, **c**, and **e** images are **h**&**e** stains, while **b**, **d**, and **f** are the correlate Masson trichrome. **a** and **b** (both 20X) are of a Simple steatosis or non-NASH NAFLD patient. **c** and **d** (both 20X) are from a “borderline” NASH patient having steatosis and pericellular fibrosis but no ballooning degeneration. **e** and **f** (40X) are from a NASH patient and show ballooning degeneration, pericellular fibrosis, and steatosis

Clinical and demographic data for the study subjects are as follows: Caucasian = 79.4%, female = 66.1%, age = 46 ± 11 years, ALT = 50.2 ± 43.3 U/L, AST = 38.8 ± 33.9 U/L and fasting serum glucose = 110.2 ± 39.6 mg/dL. Of the study group, 19 subjects had minimal histologic changes that did not meet the criteria for a pathologic diagnosis (i.e. no discernable liver disease), while 50 (73.5%) had NAFLD. Among patients that had NAFLD, 44 (64.7%) had histologic NASH. According to the NASH CRN criteria, 16 patients (23.5%) were considered to have borderline NASH while 28 (41.2%) had steatosis, lobular inflammation and unequivocal hepatocellular ballooning and would be considered definite NASH (Fig. 1).

As expected, circulating M30 and M65 were significantly higher in NASH patients as compared to those with non-NASH. In fact, serum levels of M30 were almost twice as high in NASH patients (539.1 ± 290.8 U/L vs. 287.6 ± 190.5 U/L; *p* = 0.0002), M65 was close to three times higher in NASH patients compared to all non-NASH subjects (441.2 ± 464.2 U/L vs. 162.8 ± 353.1 U/L; *p* = 0.0006). In contrast, circulating SHH failed to show differential concentrations between NASH and non-NASH subjects.

### Assessment of hepatic SHH

**Fig. 2:**
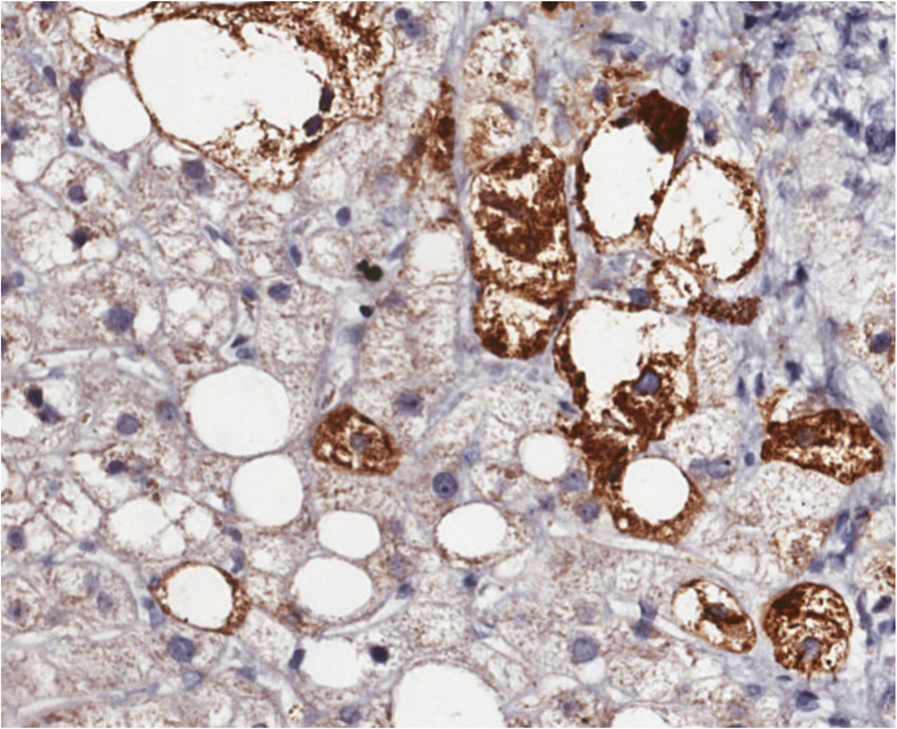
3,3′-Diaminobenzidine (DAB) staining of liver tissue using the antibody ab53281 (Brown). Staining is specific to hepatocytes undergoing ballooning degeneration

We also performed and assessed immunostaining for hepatic SHH on the liver biopsies. For further assessment of SHH signal, only slides from NAFLD subjects were considered partially because of the potential for bias caused by including 19 no-disease subjects whose biopsies lacked positive signal for SHH, and partially because examining SHH signal gradient among the NAFLD was of greater research interest. In this context, staining for SHH with ab53281 produced intense positive signal in the cytoplasm of a subset of hepatocytes undergoing ballooning degeneration, and was not seen to bind directly with Mallory-Denk bodies (Fig. 2). Additionally, a weak but consistent signal could be detected in bile duct epithelial cells, which served as a useful technical internal control. Hepatic SHH expression ranged from 0 to 8.4% of the area scanned with an average of 0.97% ± 1.8. Inter-observer variation between observers was very low (rho = 0.939, *P* < 0.0001).

Interestingly, degree of hepatic SHH signal as detected by CAM correlated with several circulating biomarkers associated with apoptosis: hepatic SHH signal positively correlated with circulating M65 (rho = 0.588, P < 0.0001), as well as circulating M30 (rho = 0.375, *P* = 0.001). Hepatic SHH signal also modestly correlated with circulating AST and ALT (rho = 0.43, *P* = 0.0004, and rho = 0.27, *P* = 0.03, respectively) (Table 1). Hepatic SHH signal did not significantly correlate with circulating SHH levels as measured by ELISA. Interestingly, the pathologist assessment of ballooning degeneration showed significant correlation to only circulating M65, M30, and AST (rho = 0.52, *P* < 0.0001; rho = 0.48, *P* < 0.0001; rho = 0.41, *P* = 0.0005, respectively). Unlike hepatic SHH signal, no correlation was detected between histopathological ballooning assessment and ALT levels.

**Table 1:** Correlations between SHH or pathology scores for pericellular fibrosis, ballooning degeneration and Circulating Analytes

Circulating Factor	SHH		Ballooning Degeneration		Pericellular Fibrosis	
	rho	*P*-Value	rho	*P*-Value	rho	*P*-Value
M65	0.588	*P* < 0.0001	0.538	*P* < 0.0001	0.548	*P* < 0.0001
M30	0.375	*P* = 0.001	0.483	*P* < 0.0001	0.525	*P* < 0.0001
AST	0.43	P < 0.0004	0.41	*P* < 0.0004	0.50	*P* < 0.0001
ALT	0.27	P < 0.03	0.18	*P* = 0.07	0.31	*P* = 0.01

**Fig. 3:**
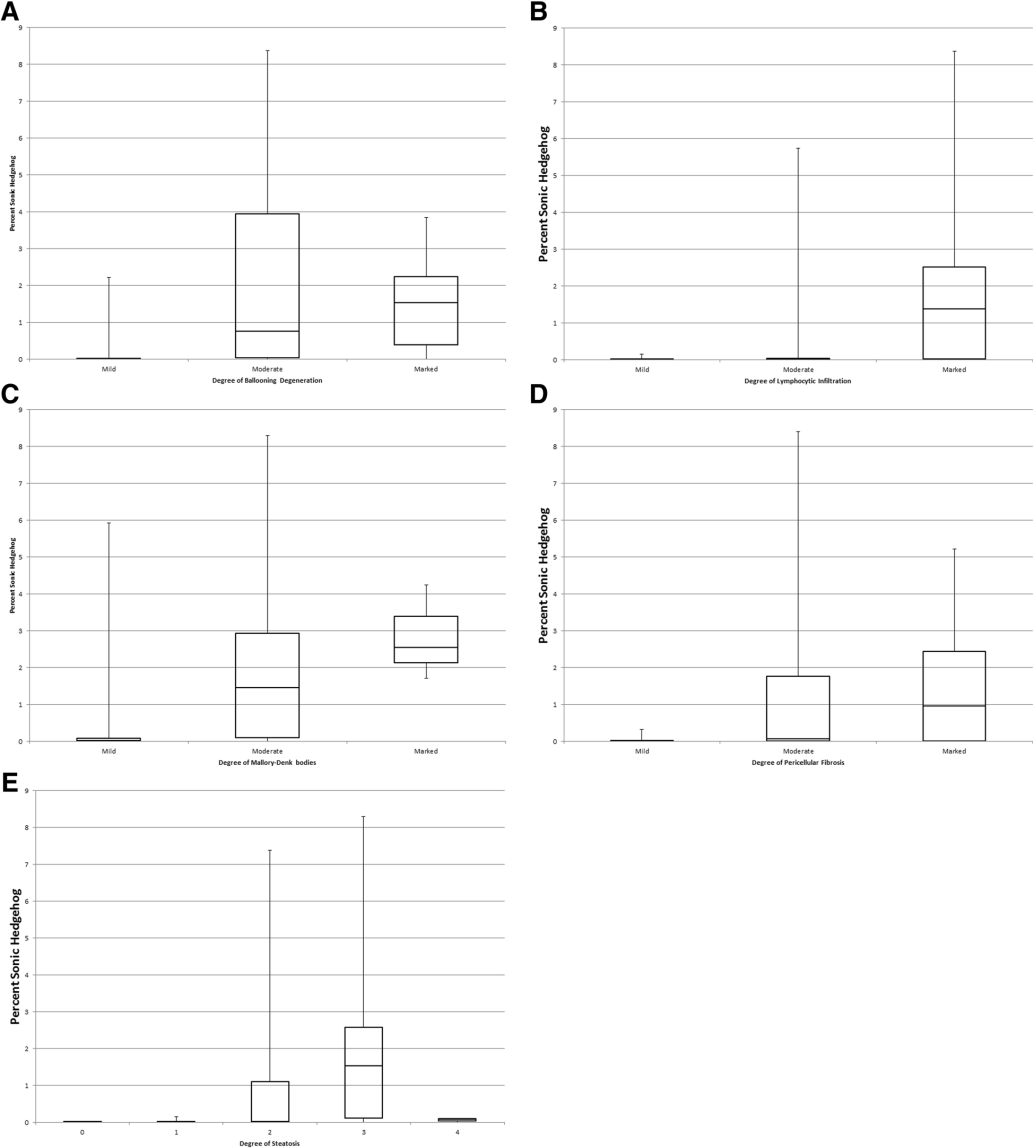
Box and Whisker plots of percent SHH vs. pathologist’s assessment of hepatic lesions: **a** Ballooning degeneration, **b** Lymphocytic infiltration, **c** Mallory-Denk bodies, **d** Pericellular fibrosis, **e** Hepatic steatosis

Hepatic SHH signal also correlated with a number of liver injury markers. Hepatic SHH signal was strongly correlated to histologic ballooning degeneration (rho = 0.62, *p* < 0.0001). Additionally, a modest but significant correlation was seen with the grade of steatosis (rho = 0.554, *p* < 0.001), Mallory-Denk bodies (rho = 0.54, p < 0.001), pericellular fibrosis (rho = 0.527, p < 0.001), and lymphocytic infiltration (rho = 0.435, *p* < 0.0002) (Table 2, Fig. 3). Although hepatic SHH signal did correlate with Mallory-Denk bodies, histologically determined ballooning degeneration much more strongly correlated with Mallory-Denk bodies (rho = 0.73, *p* < 0.0001).

**Table 2:** Correlations between SHH and Histologic Lesions

Hepatic Lesion	rho	*P*-Value
Ballooning Degeneration	0.62	*P* < 0.0001
Steatosis	0.554	*P* < 0.001
Mallory-Denk bodies	0.54	*P* < 0.001
Pericellular fibrosis	0.527	*P* < 0.001
Lymphocytic infiltration	0.435	*P* < 0.002

Although hepatic SHH immunostaining and histologic assessment of ballooning degeneration similarly correlated with the histologic assessment of hepatic pericellular fibrosis, the relationship may be more nuanced (Table 3); Degree of pericellular fibrosis among subjects based on their hepatic SHH signal and histopathological assessment of ballooning degeneration was evaluated (Table 3). Higher number of patients with mild/undetectable ballooning degeneration had mild/undetectable pericellular fibrosis (*N* = 24), when compared to those with the lowest SHH tertile (*N* = 16). However, larger number of patients had marked pericellular fibrosis in the highest SHH tertile (*N* = 12) when compared to histopathological assessment of marked ballooning degeneration (*N* = 5). Further, logistic modeling indicated hepatic SHH signal and presence of diabetes were both independent predictors of advanced fibrosis (portal fibrosis> 2 and pericellular fibrosis scores > 2: OR = 1.986, *p* = 0.01, and OR = 3.280, *p* = 0.03, respectively).

**Table 3:** Distribution of Patients by SHH tertile or assessment of ballooning degeneration and degree of pericullar fibrosis

Degree of Pericellular Fibrosis	SHH Tertile			Degree of Ballooning Degeneration		
	1st	2nd	3rd	Mild	Medium	Marked
Mild	16	9	0	24	0	1
Medium	5	8	7	8	10	2
Marked	4	4	12	6	9	5

## Discussion

The application of computer assisted image analysis with morphometric tools can provide accurate measurements of pathologic features of liver biopsy on a continuous scale, as opposed to a categorical one, which will allow broader statistical analysis and comparisons between studies. The intent of this pilot study was to assess if the benefits of computer-assisted digital image analysis could be realized in the measurement of ballooning degeneration, a key pathologic component of NASH, by quantification of SHH detection.

Our data shows significant correlation between the degrees of hepatic SHH signal and histopathologic lesions (ballooning degeneration and fibrosis). Correlations were also seen between CAM measured hepatic SHH signal and circulating forms of the epithelial cell structural protein cytokeratin18 associated with the progressive NAFLD (M30 and M65). These data warrant further validation of CAM measured hepatic SHH signal as a tool to more accurately diagnose and grade NASH. The close association between pericellular or advanced fibrosis and hepatic SHH quantification by CAM may lead to a more useful measure to parse patient populations regarding clinically important outcomes.

Though measurement of circulating cytokeratin 18 by M65 and its caspase degradation product by M30 are correlated with SHH staining, approximately half of the patients with had very low or negative SHH by CAM, suggesting that ballooning is not a direct source of these biomarkers and indicating that blood levels do not necessarily indicate marked histologic ballooning.

Although this research was not intended to explore the role of SHH in the pathogenesis of NASH, our study may offer some insight for future studies. The antibody used to detect SHH in this study (ab53281) binds in the C-terminus region of the Pro-SHH peptide, detecting either the full length pro-SHH peptide or the 27 kDa C-terminal fragment (SHH_c_). The measurable accumulation of either of these products within a subset of hepatocytes undergoing ballooning degeneration is interesting, especially given that attempts to detect the cleaved 19 kDa N-terminal fragment (using SC-9024) on serial sections were unsuccessful (Data not shown). Notably, no correlations were found between circulating serum SHH and hepatic SHH signal. This suggests the possibility of derangements in SHH post-translational modification in ballooning hepatocytes, perhaps in cholesterol addition / cleavage process of full length SHH into N and C fragments, or the proteasome degradation of SHH_c_. If so, the accumulation of SHH or SHH_c_ within ballooning hepatocytes could theoretically be a marker for those NAFLD patients in danger of progressive fibrosis.

Our study does have a few limitations. Although the sample size relatively small, we believe that the results of this pilot study can inform larger studies to validate the role of SHH staining in NASH. In addition, evaluation of any hypotheses regarding the role of SHH in the pathogenesis of NASH or fibrosis, or of its predictive value will require further experimentation over multiple time points and stages of disease.

## Conclusion

In summary, computer assisted quantitation of hepatic SHH correlates with liver lesions and circulating analytes associated with NASH. Although a larger patient population with biopsy specimens from multiple times will be needed to truly assess SHH as a grading and staging tool for NALFD, our findings are a step toward the ultimate goals of increasing the resolution of the liver biopsy reading and eliminating inter- and intra-observer variability as it pertains to ballooning assessment and NAFLD diagnosis.

## Abbreviations

ALT: Alanine Aminotransferase
AST: Aspartate Aminotransferase
CAM: Computer assisted morphometry
CK18: Cytokeratin 18
DAB: 3,3′-Diaminobenzidine
ELISA: Enzyme-linked Immunosorbent Assay
IHC: Immunohistochemistry
NAFLD: Non-alcoholic fatty liver disease
NASH CRN: Clinical Research Network in Nonalcoholic Steatohepatitis
NASH: Non-alcoholic steatohepatitis
SHH: Sonic Hedgehog
SHHc: Sonic Hedgehog C-terminal fragment
